# Surgical Emergencies among Gynecological Surgeries in a Tertiary Care Center: A Descriptive Cross-sectional Study

**DOI:** 10.31729/jnma.5888

**Published:** 2020-12-31

**Authors:** Indira Acharya, Sumana Thapa

**Affiliations:** 1 Department of Gynecology and Obstetrics, Shree Birendra Hospital, Chhauni, Kathmandu, Nepal

**Keywords:** *ectopic pregnancy*, *emergency*, *gynecological*, *obstetrics*

## Abstract

**Introduction:**

The management of gynecological emergencies is essential for the preservation of the life of affected woman, her sexual functions and fertility particularly in disease conditions that threaten her life. The main objective of the study is to determine the proportion of the surgical emergencies among gynecological surgeries in a tertiary care center.

**Methods:**

This is a descriptive cross-sectional study conducted in the department of gynecology and obstetrics in Shree Birendra Hospital, Kathmandu, Nepal from April 2013 till March 2017. Ethical approval was taken from the Institutional Review Committee (IRC) in November 2019. This study was conducted among 515 gynecological surgeries by using convenience sampling methods. Point estimate at 95% Confidence Interval was calculated along with frequency and proportion for binary data. Data were analyzed using EXCEL software.

**Results:**

In our study, the proportion of surgical emergencies among total gynecological surgeries performed in the department of gynecology and obstetrics in Shree Birendra Hospital was 120 (23.30%). The highest number of surgical emergencies was observed in the age group of 20-29 years old, followed by less than 19 years of old age group. Ectopic pregnancy accounting for 85 (70.83%) is found to be the most common surgical emergencies in our study. Out of all surgical emergency cases, most of them underwent salpingectomy 65 (54.16%) followed by salpingectomy with tubal ligation 20 (16.16%).

**Conclusions:**

Surgical emergencies among gynecological surgeries are found to be in greater proportion in the department of gynecology and obstetrics in Shree Birendra Hospital. Ectopic pregnancy accounted for more than half of the diagnoses in this study.

## INTRODUCTION

The occurrence of surgical emergencies in the department of gynecology and obstetrics is expected in everyday life.^1^ The management of such emergencies is essential to preserve life, sexual function and fertility of the affected woman.^2^ The major challenge in regard to such emergencies is the difficulty in evaluating the women in their reproductive age group and elderly women.^3^ Ultrasound helps in the early assessment of the patients with gynecological pathology.^4^

Our study is conducted in order to determine the burden of surgical emergencies in the department of gynecology and obstetrics in a tertiary care center. It is important to address such emergencies on time because the delay in diagnosis and treatment may lead to worse outcome. Early and prompt diagnosis help in their timely management.

The aim of our study is to determine the proportion of the surgical emergencies among gynecological surgeries in a tertiary care center.

## METHODS

A descriptive cross-sectional study was conducted in the department of gynecology and obstetrics in Shree Birendra Hospital, Kathmandu, Nepal from April 2013 till March 2017. Ethical approval was taken from the Institutional Review Committee (IRC) in November 2019. The study was conducted among 515 gynecological surgeries presenting to the department of gynecology and obstetrics in Shree Birendra Hospital.

The sample size was calculated using the following formula,

$$\begin{array}{ll} \text{n} & \text{= }{\text{Z}}^{\text{2}}\times \text{p}\times \text{(1}-\text{p)}/{\text{e}}^{\text{2}}\\ & \text{= }{\left(1.96\right)}^{\text{2}}\times 0.50\times 0.50/{(\text{0.07)}}^{\text{2}}\\ & \text{= }196 \end{array}$$

Where,

n = required sample size,
Z = 1.96 for Confidence Interval at 95%,
p = Prevalence, 50% (for maximum sample size)
e = margin of error as 7%

Since we used the convenience sampling method, we have doubled our sample size i.e. 196 x 2 = 392 to avoid the bias associated with the sampling. Taking a non-response rate of 10%, the sample size becomes 432.

$$\begin{array}{ll} \text{N} & \text{= 392}+\text{10\% of }392\\ & \text{= }392+39.2\\ & \text{= }431.2\\ & =432 \end{array}$$

We took a total of 515 gynecological surgeries in our study using convenience method of non-probability sampling.

All cases requiring laparotomy were included in the study. Cases requiring conservative management and suction and evacuation were excluded. Immediate resuscitation followed by detailed history taking, physical examinations, lab investigations including ultrasonography had been done for diagnosis and appropriate management were done as per the existing protocol for all patients. Data were analyzed using EXCEL software.

## RESULTS

In our study, the prevalence of surgical emergencies among total gynecology surgeries performed in the gynecology and obstetrics department of Shree Birendra Hospital was 120 (23.30%).

The highest number of surgical emergencies was observed in the age group of 20-29 years old, followed by less than 19 years of old age group (Table 1).

**Table 1:** Age wise distribution of surgical emergencies.

Age group	n (%)
<19 years	29 (24.16)
20-29 years	69 (57.5)
30-39 years	15 (12.5)
>40 years	7 (5.83)
Total	120 (100)

The most common surgical emergencies cases were diagnosed as Ectopic pregnancy 85 (70.83%) followed by Twisted/ruptured ovarian cyst 26 (21.66%) (Table 2).

**Table 2:** Distribution of surgical emergencies based on diagnosis.

Diagnosis	n (%)
Ectopic pregnancy	85 (70.83)
Twisted/ruptured ovarian cyst	23 (21.66)
Ovarian cyst with pregnancy	7 (5.83)
Others	2 (1.66)
Total	120 (100)

Out of those surgical emergency cases, most of them underwent salpingectomy 65 (54.16%) followed by salpingectomy with tubal ligation 20 (16.16%) (Table 3).

**Table 3:** Distribution of surgical emergencies based on procedures of management.

Procedures	n (%)
Salpingectomy	65 (54.16)
Salpingectomy with tubal ligation	20 (16.16)
Salpingo-oophorectomy	18 (15)
Ovarian cystectomy	15 (12.5)
Oophorectomy/ovarian cystectomy	2 (1.66)
Total	120 (100)

Among those 120 surgical emergency cases, most of them are multipara 73 (60.83%) (Table 4).

**Table 4:** Distribution of surgical emergencies based on parity.

Parity	n (%)
Nullipara	10 (8.33)
Primipara	37 (30)
Multipara	73 (60.83)
Total	120 (100)

## DISCUSSION

Gynecological emergencies are life threatening conditions that required prompt attention to save life. During the period of 5 years a total of 120 cases of emergency laparotomy were done which is almost similar to 118 cases in 5 years study Barat et al.^5^

The most common surgical emergency among gynecological surgery is ectopic pregnancy in this study. A total of 85 cases of ectopic pregnancies were seen in this study. A total of 192 cases of ruptured ectopic pregnancies were seen in the study done for 2 years duration at BPKIHS.^6^ The number of ectopic pregnancy in this study is higher in comparison with 36 cases of ectopic pregnancy seen in five years in a study at Nepal Medical College Teaching Hospital.^7^ The number of ectopic pregnancy in this study is lower in comparison with 174 cases seen in five years in a study done at Tribhuwan University Teaching Hospital.^8^ A total of 167 cases of ectopic pregnancy in five years by another study at TUTH,^9^ and 88 cases in two years in a study at B and B Hospital Kathmandu were seen.^10^ The reason for less number of cases at SBH might be that it is the hospital serving for only army personnels and army families.

The results of this study supports the results by several studies that showed ectopic pregnancy as the most common cause for emergency laparotomy which is comparable with findings in a teaching hospital in Nigeria.^11,12^

A study however showed that Pelvic Inflammatory Disease (PID) was the most common gynecological emergency in their district hospital at Kasur, Pakistan.^13^ Abortions are the most common gynecological emergency at Bayero University/Aminu Kano Teaching Hospital, Kano, Kano State Nigeria.^14^

In our study, the most common presenting complaint was pain in the lower abdomen followed by bleeding per vagina. Similar to our study, the presenting complaints were pain in the abdomen followed by bleeding per vagina in the study done in BPKIHS.^1^ A obstetric and gynecologic emergencies among pregnant study in Turkey also showed that abdominopelvic pain was the most common complaint followed by vaginal bleeding.^15^ A study in Iran showed pain in the supra pubic region followed by with nausea/vomiting as presenting complaints.^5^

In this study about 54.16% of the patients underwent only salpingectomy, which is the most common surgery done for ectopic pregnancy. Nearly 16.16% patients underwent salpingectomy with tubal ligation after taking consent from those patients willing to undergo permanent sterilization. About 83.9% of the patients underwent salpingostomy for the management of ectopic pregnancy in a tertiary hospital in Iran.^5^

In this study twisted/ruptured ovarian cyst account for 21.66%. A study done in the Department of Surgery, Nawaz Sharif Medical College, Gujrat showed ruptured ovarian cyst as 36.7%.^16^

The findings of our study cannot be generalized to the whole population of Nepal as this study was conducted in a single hospital at the capital city of Nepal.

## CONCLUSIONS

The findings of our study were nearly similar to the findings from other institutions in terms of surgical emergencies in gynecology. Ectopic pregnancy and salpingectomy are the most common cause and surgery for acute obstetric and gynecologic emergencies in a tertiary care hospital. Evaluating and counseling the women at high risk of ectopic pregnancy could be beneficial. A large prospective study is needed in order to raise awareness regarding acute lower abdominal pain and the availability of gynecologists for the management of acute obstetric and gynecologic emergencies.
